# P06-02 Physical activity levels of citizens in Dubrovnik, Croatia

**DOI:** 10.1093/eurpub/ckac095.087

**Published:** 2022-08-29

**Authors:** Dario Škegro, Zrinko Čustonja

**Affiliations:** Department of General and Applied Kinesiology, University of Zagreb Faculty of Kinesiology, Zagreb, Croatia

**Keywords:** Physical activity, Dubrovnik, Croatia, Sport

## Abstract

**Background:**

One of the most important areas of interest in the world today and throughout history is health. Numerous investigations are confirming positive effects of physical activity on health and this is the reason why physical activity and exercise should be measured as often as possible. Croatia as the youngest member state in European Union does not have excellent results in the levels of physical activity among its population. In this research the aim was to assess the levels of physical activity in the city of Dubrovnik and compare it to the levels of Physical activity in Croatia and European Union.

**Methods:**

Participants in this investigation were 670 adult citizens of the City of Dubrovnik, Croatia. Levels of physical activity was measured using Eurobarometer questionnaire. Descriptive statistical parameters Mean and Standard deviation same as Statistical Difference between the values in Dubrovnik, Croatia and European Union were calculated using statistical program package Statistica.

**Results:**

Overall results show significantly better situation in Dubrovnik when comparing with the results measured on national level and on European Union level. While, according to the Eurostat's analysis in 2017, there is 46% of citizens of European Union and even 56% of citizens in Croatia that never exercise or play sports in the City of Dubrovnik there is only 17,31% of citizens that never exercise or doing sports. The most important reasons for doing physical exercise or sport (motivators) among citizens of Dubrovnik is improvement of health (45%) and improvement of physical appearance (30%). Almost half of the sample (47%) pointed out the lack of time as the main barrier in doing physical exercise or sport.

**Conclusions:**

This investigation shows good way to assess the information on levels of physical activity of the citizens and accordingly creating a public policy on further development and improvement of this important area. This investigation is part of document called Strategic development of Sport and Sport's Infrastructure in Dubrovnik and it reveals encouraging data for this Croatian city.

